# Features of severe asthma response to anti-IL5/IL5r therapies: identikit of clinical remission

**DOI:** 10.3389/fimmu.2024.1343362

**Published:** 2024-01-23

**Authors:** Giovanna Elisiana Carpagnano, Andrea Portacci, Santi Nolasco, Aikaterini Detoraki, Alessandro Vatrella, Cecilia Calabrese, Corrado Pelaia, Francesca Montagnolo, Giulia Scioscia, Giuseppe Valenti, Maria D’Amato, Maria Filomena Caiaffa, Massimo Triggiani, Nicola Scichilone, Claudia Crimi

**Affiliations:** ^1^ Department of Translational Biomedicine and Neuroscience, Institute of Respiratory Disease, University “Aldo Moro”, Bari, Italy; ^2^ Respiratory Medicine Unit, Policlinico “G. Rodolico-San Marco” University Hospital, Catania, Italy; ^3^ Department of Clinical and Experimental Medicine, University of Catania, Catania, Italy; ^4^ Division of Internal Medicine and Clinical Immunology, Department of Internal Medicine and Clinical Complexity, Azienda Ospedaliera Universitaria Federico II, Napoli, Italy; ^5^ Department of Medicine, Surgery and Dentistry, University of Salerno, Salerno, Italy; ^6^ Unitá Operativa (UO) Clinica Pneumologica SUN, Dipartimento Pneumologia ed Oncologia, Azienda Ospedaliera Specialistica dei Colli, Napoli, Italy; ^7^ Department of Health Sciences, University “Magna Graecia” of Catanzaro, Catanzaro, Italy; ^8^ Department of Medical and Surgical Sciences, University of Foggia, Foggia, Italy; ^9^ Allergology and Pulmonology Unit, Provincial Outpatient Center of Palermo, Palermo, Italy; ^10^ Unitá Operativa Semplice Dipartimentale (UOSD) Malattie Respiratorie “Federico II”, Ospedale Monaldi, Azienda Ospedaliera (AO) Dei Colli, Naples, Italy; ^11^ Department of Medical and Surgical Sciences, School and Chair of Allergology and Clinical Immunology, University of Foggia, Foggia, Italy; ^12^ Division of Allergy and Clinical Immunology, University of Salerno, Salerno, Italy; ^13^ Division of Respiratory Diseases, Department of Health Promotion Sciences, Maternal and Infant Care, Internal Medicine and Medical Specialties (PROMISE), University of Palermo, Palermo, Italy

**Keywords:** severe asthma, anti-IL5, anti-IL5 receptor, clinical remission, biologics, FEV_1_, exacerbations, corticosteroids

## Abstract

**Introduction:**

Clinical remission (CliR) achievement has been recognized as a new potential outcome in severe asthma. Nevertheless, we still lack a detailed profile of what features could better identify patients undergoing clinical remission. In this study, we aim to address this issue, tracing a possible identikit of patients fulfilling remission criteria.

**Methods:**

We enrolled 266 patients with severe eosinophilic asthma (SEA) treated with a 12-month course of anti-IL5/IL5 receptor (IL5r) monoclonal antibodies. Patients with no exacerbation, OCS withdrawal, ACT ≥ 20 and FEV_1_ ≥ 80% after 1 year of biologic treatment were classified as in clinical remission.

**Results:**

30.5% of the enrolled patients achieved remission after biologic administration. CliR group showed a lower number of baseline asthma exacerbations and better lung function parameters, with a trend for higher ACT scores and a less frequent history of a positive skin prick test. CliR achievement was unlikely in presence of a higher BMI, a positive skin prick test, an increased number of asthma exacerbations before biologic treatment, anti-muscarinic administration, and a previous diagnosis of EGPA, bronchiectasis or osteoporosis. In contrast, a better lung function, an increased blood eosinophilic count, the presence of chronic rhinosinusitis with nasal polyps and a more frequent use of reliever therapy predicts remission development. Changes in exacerbations number, OCS use, ACT scores and FEV_1_% between remittent and non-remittent patients arise at specific follow up timepoints and are positively associated with CliR achievement.

**Discussion:**

anti-IL5/IL5r biologics can induce CliR in a proportion of patients with SEA. Patients achieving remission demonstrate specific clinical, functional and inflammatory features, as well as a specific moment of improvement in all the CliR items.

## Introduction

In the past years, the primary focus in asthma care was centered around controlling symptoms using appropriate inhaled corticosteroid (ICS) therapy and, when needed, oral corticosteroid (OCS) (1). However, the advent of biologics has introduced a new era in asthma treatment, enabling targeted and personalized approaches. As a result of this therapeutic evolution, many patients with severe asthma experienced a significant reduction in exacerbations and OCS administration and improvement in their lung function as well as their quality of life.

These positive outcomes have given rise to a more ambitious aim: achieving potential remission of the disease. The concept of remission, similarly to other chronic conditions, is increasingly becoming widespread, and it should be regarded as a new cornerstone of asthma management. This notion is supported by the observation that spontaneous remission occurs in varying percentages (ranging from 5% to 69%) of children during their transition into adolescence and adulthood (2). Based on the recognition of this natural occurrence, some authors have extended this concept, identifying monoclonal antibodies as potentially able to induce clinical remission (CliR) in a subset of patients with type-2 severe asthma. Recently, several definitions of CliR have been proposed by different research groups, according to which subset of criteria was considered to classify patients as remittent. Menzies-Gow et al, in a three round Delphi survey involving many experts in the field of severe asthma, defined the achievement of CliR after at least 12 months of follow up according to four main criteria: 1) the absence of relevant symptoms or asthma exacerbations; 2) the complete cease of systemic corticosteroid treatments; 3) the improvement and stabilization of lung function; 4) the agreement between patients and health care practitioners on remission achievement (3). The presence of CliR along with the absence of signs related to asthma inflammation or bronchial hyperresponsiveness would then define a complete on-treatment remission. Later, Canonica and his collaborators from the Severe Asthma Network Italy (SANI) provided another definition of CliR (4), with the cessation of OCS therapies for at least 12 months as a crucial element for CliR achievement and the absence of symptoms, exacerbations, and a documented lung function stability as additional criteria to separate a partial CliR (two out of three criteria met) from a complete CliR (all three criteria met). Following this path, several studies adopted a wide range of different parameters and cut-offs for CliR definition (5–7), also revealing that patients undergoing biologic therapies and achieving remission seem to exhibit distinctive features, such as a more pronounced T-helper 2 inflammatory profile, better baseline clinical and functional performance, and a specific subset of comorbidities that could be predictive of a good treatment response (3, 4, 7–11). Despite this evidence, we need more crucial information about what clinical, functional, and biological features could predict the achievement of CliR.

Our study aimed to conduct an extensive analysis of these factors to establish a detailed patient profile that would be associated with CliR in patients treated with anti-IL5 or anti-IL5 receptor (IL-5r) monoclonal antibodies.

## Materials and methods

### Study design

We conducted a real-life, observational, retrospective, multicenter analysis from the “Southern Italy Network on Severe Asthma Therapy”, screening patients who underwent a one-year treatment course with Mepolizumab or Benralizumab from September 2017 to March 2022.

### Study population

**Figure 1:**
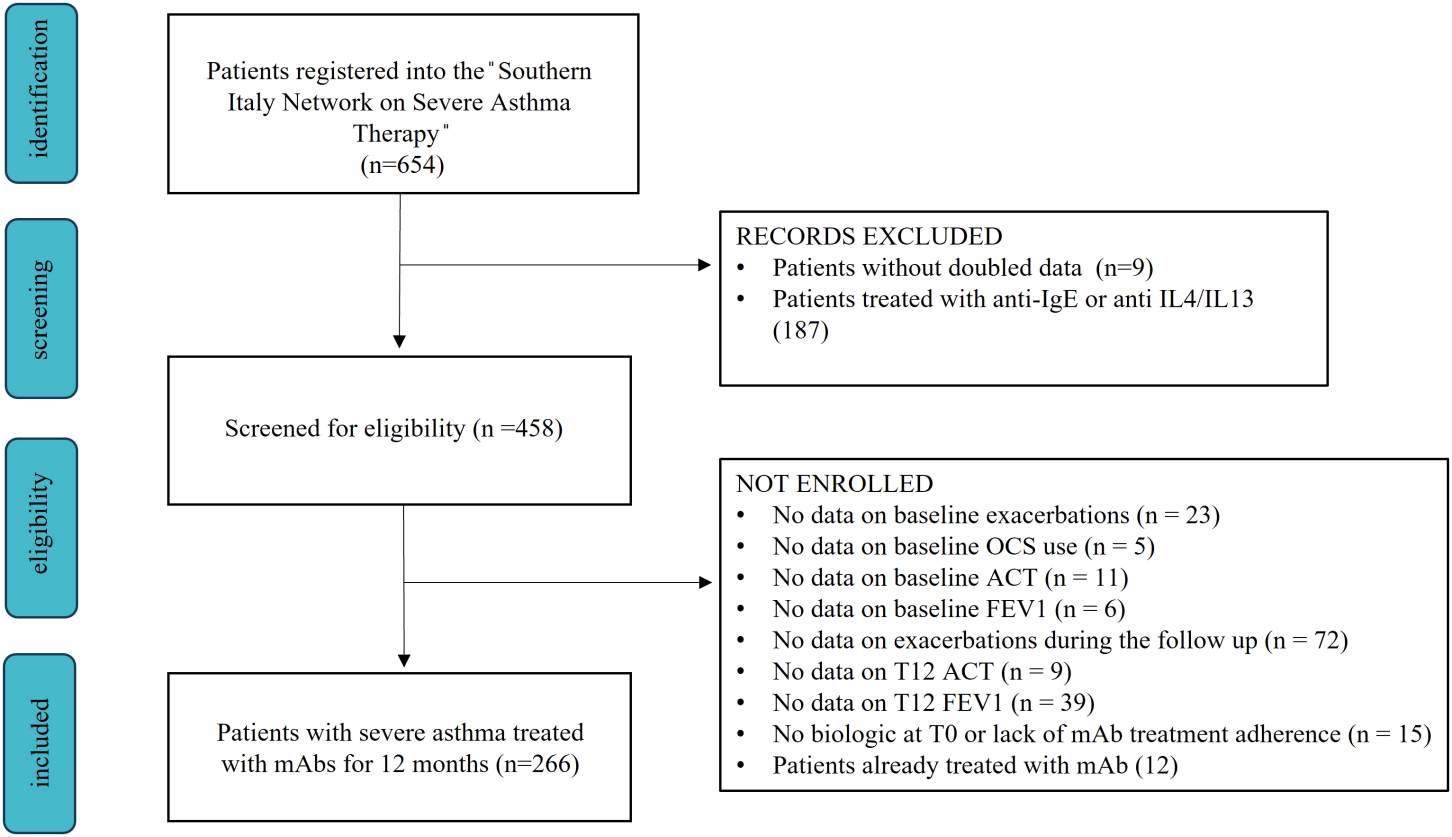
Flow chart showing the enrollment process from the “Southern Italy Network on Severe Asthma Therapy” database.

The “Southern Italy Network on Severe Asthma Therapy” includes 654 patients with severe asthma treated with monoclonal antibodies. For the final analysis, we included 266 patients with age > 18 years and severe eosinophilic asthma (SEA) treated with anti-IL5 or anti-IL5 receptor (IL-5r) monoclonal antibodies for at least 12 consecutive months (**Figure 1**). SEA diagnosis was made according to GINA recommendations and ERS guidelines (1, 12). During baseline visit (T0) we gathered anamnestic and anthropometric data, information regarding asthma exacerbation, maintenance and rescue therapies, lung function and Type-2 inflammatory biomarkers.

Asthma Control Test (ACT) and Test of Adherence to Inhalers (TAI) were used to assess symptoms control and inhaled treatment adherence, respectively. In case of poor medication adherence (TAI < 50 points), patients were re-trained on the use of their inhaler and encouraged to fulfill the prescribed therapy. Anti-IL5/IL5r biologics were prescribed according to the following criteria:

- Mepolizumab 100 mg once a month in patients with blood eosinophilic count (BEC) > 150 cells/mm3 (and a single BEC > 300 cells/mm3 in the last year) and the need of a treatment with OCS for at least 6 months or ≥ 2 exacerbations treated with OCS or hospitalization in the last year;
- Benralizumab 30 mg (once every 4 weeks for the first 3 doses, then every 8 weeks) in presence of a BEC ≥ 300 cells/mm3 and the need of a treatment with OCS or ≥ 2 exacerbations treated with OCS or hospitalization in the last year.

From a clinical standpoint, all the centers of the network agreed to evaluate a possible biologic discontinuation due to lack of efficacy only after a complete 1-year course of Mepolizumab or Benralizumab. For this reason, our final dataset could also include patients with a partial/poor response to biologics along with those achieving remission, allowing more generalizable results.

During the follow up, we performed visits after 1 (T1), 3 (T3), 6 (T6) and 12 (T12) months from the start of biologic treatment. CliR was defined after 1 year of biologic therapy when patients achieved no exacerbations, no OCS maintenance treatment, ACT ≥ 20 and FEV_1_ ≥ 80%.

Patients were excluded from the study in case of lack of treatment adherence, previous use of severe asthma monoclonal antibodies or missing data on items defining clinical remission (OCS administration, exacerbations, symptoms and lung function).

The study was approved by the Bari Institutional Ethics Committees (Ethical Committee number: 6313) and was conducted following the Helsinki Declaration of 1975 and the Good Clinical Practice standards. Patients signed written informed consent before enrollment.

### Statistical analysis

After assessing data distribution with Kolmogorov-Smirnov test, we compared means and standard deviations (SD) of continuous variables with normal distribution using T-test, while data exhibiting non-normal distribution were analyzed with Mann-Whitney-U as medians and interquartile ranges (IQR). Discrete variables were expressed with percentages and analyzed with Chi² or Fisher exact test. In presence of missing data (see **Supplementary Tables 1, 2**), we performed multiple imputation analysis before processing our dataset. Then, to explore which factors could be predictive of CliR achievement, we run a multivariate logistic least absolute shrinkage and selection operator (LASSO) regression, selecting variables significantly associated with the outcome. Briefly, LASSO regression, differently from other regression analyses, allows variables selection using a penalty (λ), which reduces variance and shrinks toward zero non-relevant covariates (13). Finally, we tested the robustness of our models using receiver operating characteristics (ROC) curves with their areas under the curves (AUC). Statistical analysis was performed using SPSS Statistics 26 (IBM Corporation) and R software (version 4.0.2, R Foundation), considering a P value < 0.05 as statistically significant.

## Results

### Baseline features

Our study analyzed 266 patients with severe asthma (see **Figure 1**), 154 were treated with Mepolizumab and 112 with Benralizumab. Baseline characteristics of the enrolled patients are reported in **Table 1**. Our study population was predominantly female (65%), with a mean age of 58 years and a mean BMI of 26.3 kg/m^2^. Most patients had no smoking history (66.5%) with only 5.6% of them classified as current smokers. The most reported comorbidities were chronic rhinosinusitis with nasal polyposis (CRwNP, 50%) and gastroesophageal reflux disease (GERD, 35.3%). Nearly all patients had at least one asthma exacerbation in the year before the start of biologic treatment, 31.6% of whom required an access to emergency department (ED). Furthermore, our cohort exhibited a mean ACT score of 13.4 points and a frequent use of OCS as maintenance treatment (75.6%), with a median dose of 12.5 mg/day. Considering T-helper 2 biomarkers, fraction exhaled nitric oxide (FeNO), blood eosinophilic count (BEC) and total immunoglobulin E (IgE), median values were 36 ppb, 645 cell/mcl and 155.4 U/lt, respectively. During the follow-up, the use of biologics improved all the explored features (see **Supplementary Table 3**; **Supplementary Figure 1**). Asthma exacerbation frequency, accesses to emergency departments due to respiratory symptoms worsening and OCS administration dropped from T0 to T12 (P < 0.0001), along with FeNO (P = 0.0007) and BEC reduction (P < 0.0001). Oppositely, ACT scores and lung function rose after the start of biologic treatment (see **Supplementary Figures 1, 2**), confirming the improvement granted by anti-IL5/IL5r on severe asthma symptoms. None of the enrolled patients experienced any serious adverse effect due to biologic administration, defined as any reaction leading to death, hospitalization or a persistent disability during the follow up time (14).

**Table 1:** Baseline features of the overall enrolled population according to the administered biologic treatment.

Biologic therapy	Overall	Mepolizumab	Benralizumab	P-value^*^
Patients (n)	266	155	111	
Age (Years, Median, IQR)	58 [48-65]	59 [51-65]	57 [46-65]	0.14
Gender (Male/Female, %)	35/65 (93/173)	33.5/66.5 (52/103)	36.9/63.1 (70/41)	0.6
BMI (Days, Median, IQR)	26.3 [23.4-29.1]	27 [23.6-29.8]	25.7 [22.8-28.1]	**0.02**
Smoke habits (%, n)				0.39
o Current smoker o Former smoker o No smoker	5.6 (15) 27.8 (74) 66.5 (177)	5.2 (8) 31 (48) 63.9 (99)	6.3 (7) 23.4 (26) 70.3 (78)	
Age of asthma onset (Years, Median, IQR) Asthma duration (Years, Median, IQR) Positive skin prick test (%, n)	37 [25-46] 19 [10˗29] 56.8 (151)	37 [25-48] 18 [10˗27] 62.6 (97)	37 [24-44] 19 [13-29] 48.6 (54)	0.42 0.47 **0.025**
Comorbidities (%, n)				
o EGPA o CRwNP o Bronchiectasis o GERD o OSAS o Depression o Urticaria o Atopic dermatitis o Osteoporosis	5.6 (15) 50 (133) 18.8 (50) 35.3 (94) 6.8 (18) 17.7 (47) 4.5 (12) 4.9 (13) 10.9 (29)	6.5 (10) 47.1 (73) 19.4 (30) 32.3 (50) 7.7 (12) 20.6 (32) 3.2 (5) 3.2 (5) 9.7 (15)	4.5 (5) 54.1 (60) 18 (20) 39.6 (44) 5.4 (6) 13.5 (15) 6.3 (7) 7.2 (8) 12.6 (14)	0.6 0.32 0.87 0.24 0.62 0.14 0.25 0.16 0.55
Exacerbations in the past year (%, n) Exacerbations in the past year (Median, IQR) Access to ED (%, n) ACT baseline (Mean, SD)	96.2 (256) 4 [3-6] 31.6 (84) 13.4 ± 4	96.1 (149) 5 [3-7] 29.7 (46) 13.1 ± 4	96.4 (107) 4 [3-6] 34.2 (38) 13.9 ± 4	0.99 **0.048** 0.5 0.75
Asthma treatment				
o LAMA (%, n) o Reliever (%, n) o Reliever use (Median, IQR#) o LTRA (%, n) o OCS (%, n) o OCS dose (Median, IQR)	69.9 (186) 59 (157) 1.5 [0-3] 44.4 (118) 75.6 (201) 12.5 [5-25]	65.2 (101) 58.7 (91) 1 [0-3] 48.4 (75) 74.2 (115) 12.5 [6.2-25]	76.6 (85) 59.5 (66) 2 [0-2] 38.7 (43) 77.5 (86) 10 [5-25]	0.06 0.99 0.85 0.13 0.57 **0.01**
Lung function				
o FEV_1_ (%, Mean, SD) o FEV_1_ (lt, Mean, SD) o FVC (%, Mean, SD) o FVC (lt, Mean, SD) o FEV_1_/FVC (Mean, SD) o FEF_25-75_ (Median, IQR)	69.8 ± 22.1 1.9 ± 0.8 84.5 ± 20.5 2.8 ± 1 67 ± 12.3 39 [25-56.2]	70.9 ± 22.4 1.9 ± 0.9 85.6 ± 20.6 2.8 ± 1 67.2 ± 12.4 40.3 [25-56.9]	68.3 ± 21.7 1.9 ± 0.7 83 ± 20.4 2.8 ± 0.9 66.8 ± 12.1 37 [24-56]	0.1 0.33 0.78 0.3 0.57 0.83
FeNO (ppb, Median, IQR) BEC (cells/mcl, Median, IQR) Total IgE (IU/mL, Median, IQR)	36 [19.4-59.5] 645 [420-920] 155.4 [62-340]	33.5 [16-60.7] 600 [400-950] 179 [60-351]	40 [24-59] 670 [453-900] 119 [65.7-318.8]	0.15 0.45 0.62

IQR, Interquartile Range; EGPA, Eosinophilic Granulomatosis with Polyangiitis; CRwNP, Chronic Rhinosinusitis with Nasal Polyps; BMI, Body Mass Index; ED, Emergency Department; OCS, Oral Corticosteroids; GERD, Gastroesophageal reflux disease; OSAS, Obstructive Sleep Apnea Syndrome; ED, Emergency Department; ACT, Asthma Control Test; LAMA, Long-Acting Muscarinic Antagonists; LTRA, Leukotriene receptor antagonist therapy; SD, Standard Deviation; FEV_1_, Forced Expiratory Volume; FVC, Forced Vital Capacity; FEF, Forced Expiratory Flow; FeNO, Fractional Exhaled Nitric Oxide; BEC, blood eosinophil count. *P-values are related to biologics comparisons. Bold p-values are those statistically significant (p < 0.05).

As regard main differences according to the administered biologic treatment, patients receiving Mepolizumab reported a higher BMI, were more frequently positive to the skin prick test (see **Table 1**, P = 0.025) and had a slightly increased number of exacerbations in the year before the enrollment (P = 0.048) requiring higher dosed of OCS (P = 0.01).

### Characteristics of remittent patient

**Figure 2:**
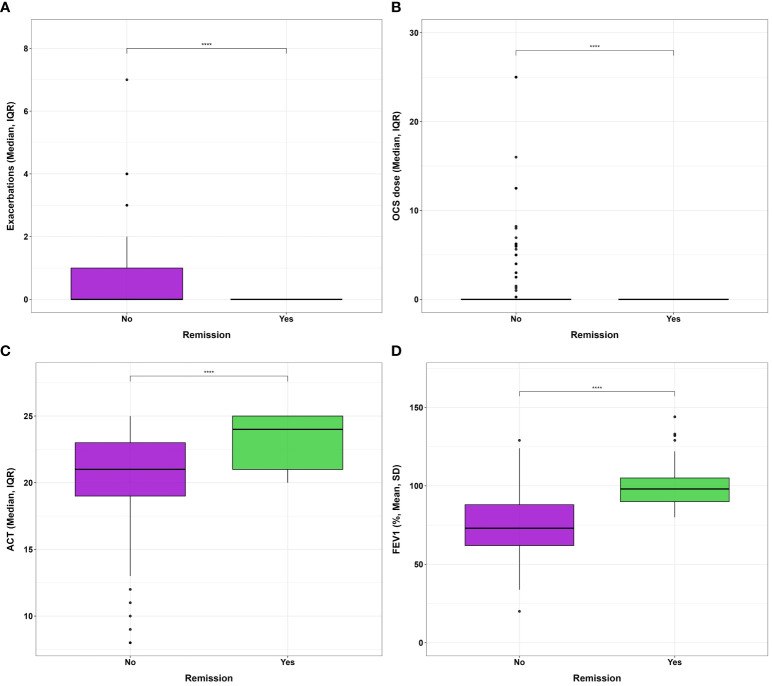
Differences in **(A)** exacerbation number, **(B)** oral corticosteroid administered dose, **(C)** ACT and **(D)** FEV_1_% after 1 year of biologic therapy according to clinical remission achievement. **** P<0.0001.

**Figure 3:**
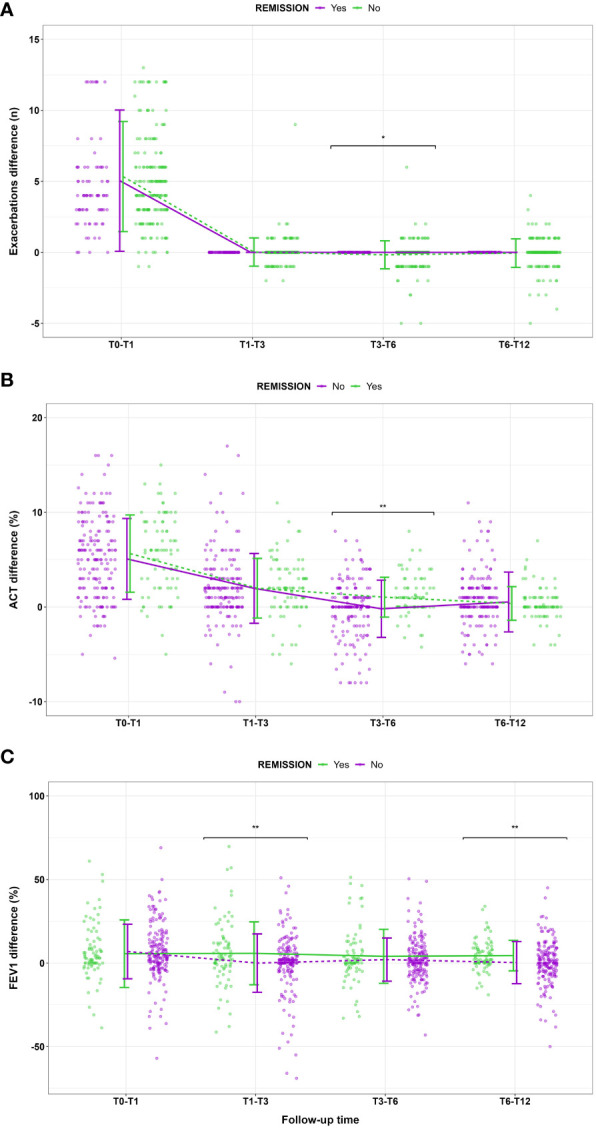
Main changes in **(A)** exacerbation number, **(B)** ACT score and **(C)** FEV_1_% according to clinical remission achievement at different follow up timepoints. Data are presented as means and standard deviations for graphical purposes. * P<0.05; **P<0.01.

Starting from the proposed criteria for CliR definition (3, 4), 30.5% of the enrolled patients achieved this outcome, while 64.7% fulfilled ≥ 3 criteria and 84.2% at least 2 items. Notably, 77.3% of non-CliR patients still achieved at least 2 remission criteria, confirming the substantial impact of biologic treatments even in non-remittent patients (see **Supplementary Table 4**). Patients belonging to CliR group revealed a better baseline clinical status (see **Table 2**). Indeed, those achieving remission showed higher mean ACT scores (P = 0.07), reporting less exacerbations (P = 0.04), a less frequent prescription of anti-muscarinic (P = 0.014) and a better lung function at baseline, as demonstrated by higher FEV_1_, FVC and FEF_25-75_ values (P < 0.0001). Baseline clinical features of patients achieving CliR showed no substantial differences according to the choice of biologic treatment (See **Supplementary Table 5**), while Non-CliR patients receiving Mepolizumab had higher BMI (P = 0.03), lower levels of FeNO (P = 0.02) and a higher number of exacerbations in the year before the enrollment (P = 0.03) requiring greater OCS doses (P = 0.03). After 12 months of anti-IL5/IL5r therapy, we found a significant ACT (P < 0.0001) and FEV_1_ (P < 0.0001) improvement in remittent patients (see **Supplementary Table 6**), which could explain the simultaneous reduction in the reliever therapy use (P = 0.02) and in LAMA administration (P = 0.03). **Table 3** shows main differences in CliR criteria according to remission status and follow up timepoints. Patients achieving CliR decreased the number of exacerbations and the use of OCS between T3 and T6 (P = 0.05, P = 0.03, respectively), also increasing their ACT scores in the same follow up phase (see **Figure 2**, P = 0.001). In contrast, FEV_1_ gap between patients achieving CliR and those without remission followed two phases, with a first increase between T1-T3 (P = 0.01) and a second one from T6 to T12 (P = 0.007, see **Figure 3**).

**Table 2:** Baseline features in patients achieving clinical remission (CliR) vs non-remittent patients (Non-CliR).

	CliR	Non-CliR	P value
Patients (%, n)	30.5 (81)	69.5 (185)	
Age (Years, Median, IQR)	57 [47.5-63.5]	58 [48.5-65]	0.26
Gender (Male/Female, %)	38.3/61.7	33.5/66.5	0.49
BMI (Days, Mean, IQR)	26.1 ± 4.9	27.1 ± 5.3	0.13
Smoke habits (%, n)			0.12
o Current smoker o Former smoker o No smoker	1.2 (1) 29.6 (24) 69.1 (56)	7.6 (14) 27 (50) 65.4 (121)	
Age of asthma onset (Years, Median, IQR) Time from asthma diagnosis (Years, Median, IQR) Positive skin prick test (%, n)	37 [25-44.5] 18 [10-27.5] 48.1 (39)	37 [24-48] 19 [10-30] 60.5 (112)	0.64 0.31 0.08
Comorbidities (%, n)			
o EGPA o CRwNP o Bronchiectasis o GERD o OSAS o Depression o Urticaria o Atopic dermatitis o Osteoporosis	6.2 (5) 58 (47) 13.6 (11) 35.8 (29) 3.7 (3) 12.2 (10) 2.5 (2) 4.9 (4) 6.2 (5)	5.4 (10) 47 (86) 21.1 (39) 35.1 (65) 8.1 (15) 20.8 (37) 5.4 (10) 4.9 (9) 13 (24)	0.78 0.11 0.17 1.00 0.29 0.16 0.36 1.00 0.13
Exacerbations (%, n) Exacerbations at 1^st^ visit (Median, IQR) Access to ED (%, n) ACT baseline (Mean, SD)	95.1 (77) 4 [3-6] 25.9 (21) 14.1 ± 4.3	96.8 (179) 5 [3-7] 34.1 (63) 13.2 ± 3.9	0.50 **0.04** 0.20 0.07
Asthma treatment			
o LAMA (%, n) o Reliever (%, n) o Reliever use (Median, IQR#) o LTRA (%, n) o OCS (%, n) o OCS dose at baseline (Median, IQR)	59.3 (48) 58 (47) 1 [0-2] 43.2 (35) 69.1 (56) 12.5 [5-25]	76.4 (138) 59.5 (110) 1 [0-3] 44.9 (83) 78.4 (145) 12.5 [5-25]	**0.014** 0.89 0.92 0.89 0.12 0.81
Lung function			
o FEV_1_ (%, Mean, SD) o FEV_1_ (lt, Mean, SD) o FVC (%, Mean, SD) o FVC (lt, Mean, SD) o FEV1/FVC (Mean, SD) o FEF_25-75_ (Median, IQR)	79.2 ± 20.6 2.2 ± 0.9 91.7 ± 18.3 3.1 ± 1.1 70.1 ± 12.4 50 [38.7-64.5]	65.7 ± 21.5 1.7 ± 0.7 81.4 ± 20.6 2.7 ± 0.9 65.7 ± 12 33 [22.9-49.5]	**<0.0001** **<0.0001** **<0.0001** **<0.0001** **0.007** **<0.0001**
FeNO (ppb, Median, IQR) BEC (cells/mcl, Median, IQR) Total IgE (IU/mL, Median, IQR)	36 [24-66] 620 [475-1058.5] 181 [65.8-337.1]	35.5 [15-52.5] 650 [410-900] 150 [61.6-343.6]	0.28 0.35 0.91

IQR, Interquartile Range; EGPA, Eosinophilic Granulomatosis with Polyangiitis; CRwNP, Chronic Rhinosinusitis with Nasal Polyps; BMI, Body Mass Index; ED, Emergency Department; OCS, Oral Corticosteroids; GERD, Gastroesophageal reflux disease; OSAS, Obstructive Sleep Apnea Syndrome; ED, Emergency Department; ACT, Asthma Control Test; LAMA, Long-Acting Muscarinic Antagonists; LTRA, Leukotriene receptor antagonist therapy; SD, Standard Deviation; FEV_1_, Forced Expiratory Volume; FVC, Forced Vital Capacity; FEF, Forced Expiratory Flow; FeNO, Fractional Exhaled Nitric Oxide; BEC, blood eosinophil count. Bold p-values are those statistically significant (p < 0.05).

**Table 3:** Change in clinical remission features during follow up timepoints.

	T0-T1	P Value	T1-T3	P Value	T3-T6	P Value	T6-T12	P Value
Exacerbations (Median, IQR)								
o Remission o Non-remission	4 [3-6] 5 [3-6.5]	0.25	0 [0-0] 0 [0-0]	1.00	0 [0-0] 0 [0-0]	**0.045**	0 [0-0] 0 [0-0]	0.93
OCS (%, n)								
o Remission o Non-remission	46.9 (38) 49.7 (92)	0.69	12.3 (10) 10.8 (20)	0.68	19.8 (16) 9.7 (18)	**0.03**	6.2 (5) 13.5 (25)	0.09
ACT (Mean, SD)								
o Remission o Non-remission	5.64 ± 4.08 5.08 ± 4.27	0.32	1.98 ± 3.15 1.97 ± 3.68	0.97	1.03 ± 2.11 -0.19 ± 3.02	**0.001**	0.38 ± 1.77 0.53 ± 3.16	0.68
Pre-BD FEV_1_ (%, Mean, SD)								
o Remission o Non-remission	5.60 ± 20.2 6.91 ± 16.3	0.58	5.82 ± 18.8 -0.03 ± 17.5	**0.01**	4.02 ± 16.2 2.05 ± 12.9	0.29	4.46 ± 9.13 0.25 ± 12.6	**0.007**

IQR, Interquartile Range; OCS, Oral Corticosteroids; ACT, Asthma Control Test; FEV_1_, Forced Expiratory Volume; SD, Standard Deviation. Bold p-values are those statistically significant (p < 0.05).

### Predictive features for CliR achievement

**Figure 4:**
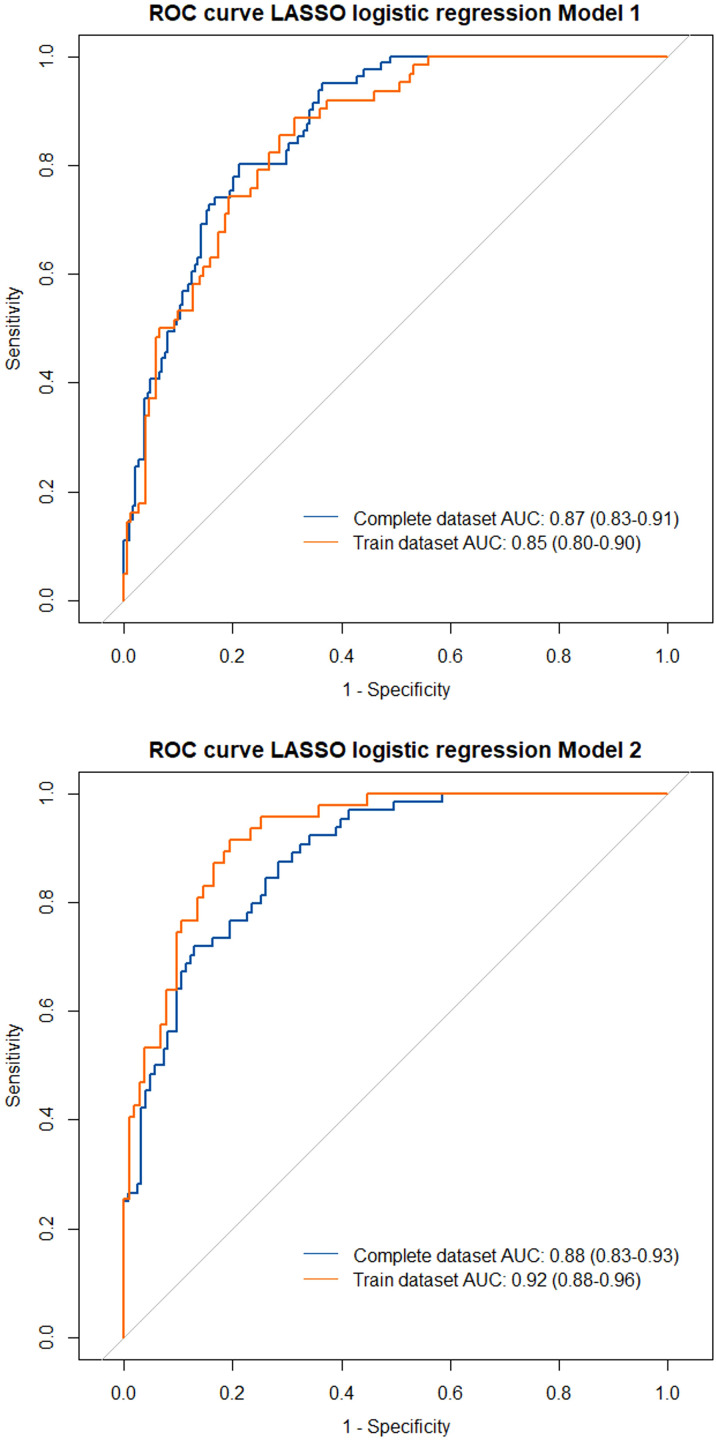
Receiver operating characteristics (ROC) curves with area under the curves (AUC) for the tested LASSO regression models. Model 1 included all the enrolled patients in the analysis, considering the complete dataset (blue line) and the training dataset of the LASSO regression (orange line). Model 2 only included patients with a recorded FeNO at baseline.

We performed a LASSO logistic regression considering several clinical, functional, and biological variables from the entire enrolled cohort (Model 1, see **Table 4**). Due to the amount of missing data at baseline, FeNO was excluded from the first regression analysis, being separately tested in a different LASSO regression model (Model 2). Model 1 revealed that patients with a higher baseline BEC, with better lung function, with comorbid CRwNP, with a more frequent administration of OCS or inhaled reliever therapy (as needed SABA or ICS-Formoterol) were more likely to attain this outcome. Moreover, changes in main remission items (exacerbations, OCS, ACT, FEV_1_) at specific follow up timepoints were all related to CliR development at T12. In contrast, the presence of a positive skin prick test, a higher Body Mass Index (BMI), a greater number of exacerbations before the start of the biologic therapy, the administration of Long-Acting Muscarinic Antagonists (LAMA), a previous diagnosis of comorbid eosinophilic granulomatosis with polyangiitis (EGPA), bronchiectasis or osteoporosis were negative predicting factors for CliR development. Model 2 did not demonstrate a significant role of FeNO on remission achievement. Finally, ROC curves analysis confirmed the robustness of our models (see **Supplementary Table 7** and **Figure 4**, Model 1 AUC = 0.87 P < 0.0001; Model 2 AUC = 0.88, P < 0.0001).

**Table 4:** LASSO logistic regression models for clinical remission.

	LASSO logistic regression	
	Model 1	Model 2
**Best lambda**	0.019	0.018
**Variables**	**Coefficients**	
Age	/	/
Gender	/	/
BMI	-0.33	/
Smoking status	/	0.64
Years from asthma diagnosis	/	/
Positive skin prick test	-0.28	-1.49
Exacerbations	-1.10	/
Exacerbations T3-T6	-9.29	-11.5
Access to ED	/	-0.26
LAMA	-0.03	/
Reliever use	0.10	1.80
LTRA	/	0.04
OCS	/	/
OCS T3-T6	0.10	1.22
ACT	/	0.39
ACT T3-T6	1.49	1.42
EGPA	-0.36	/
CRwNP	0.40	/
Bronchiectasis	-0.16	/
Depression	/	/
GERD	/	/
OSAS	/	/
Osteoporosis	-0.13	-0.78
Urticaria	/	/
Atopic dermatitis	/	/
FEV_1_%	2.23	1.82
FEV_1_ T1-T3	3.39	3.26
FEV_1_ T6-T12	0.39	1.16
FVC%	/	/
FEF_25-75_	0.83	1.34
BEC	0.69	1.81
IgE	/	0.44
FeNO	*NA*	/

Model 1 has been developed using baseline (T0) variables and some covariates expressing a significant change of specific parameters (exacerbations, OCS use, ACT, FEV_1_) during follow up timepoints. Model 2 only accounts patients with a recorded FeNO at baseline, using Model 1 covariates for the analysis.

BMI, Body Mass Index; ED, Emergency Department; LAMA, Long-Acting Muscarinic Antagonists; SABA, Short-acting beta-2 agonists; LTRA, Leukotriene receptor antagonist therapy; OCS, Oral Corticosteroids; ACT, Asthma Control Test; EGPA, Eosinophilic Granulomatosis with Polyangiitis; CRwNP, Chronic Rhinosinusitis with Nasal Polyps; GERD, Gastroesophageal reflux disease; OSAS, Obstructive Sleep Apnea Syndrome; FEV_1_, Forced Expiratory Volume; FVC, Forced Vital Capacity; FEF, Forced Expiratory Flow; BEC, blood eosinophil count.

## Discussion

Our study highlights which factors could contribute to the achievement of CliR in patients with severe asthma undergoing biologic treatment with Mepolizumab or Benralizumab. Among the enrolled population, over 30% of patients treated with Mepolizumab or Benralizumab achieved CliR at T12, a result in line with data from other real-life cohorts (7). Notably, we found no substantial differences in the remission rate (P = 0.59) and in the baseline features of patients achieving CliR according to the administered biologic (see **Supplementary Table 5**). These data probably reflect the similar clinical and functional effects of anti-IL5 and anti-IL5R following eosinophils depletion, which translates into comparable remission rates and CliR baseline features. Future prospective studies should assess whether biologic choice could represent a discriminating factor for CliR prediction.As reported in **Table 2**, remittent patients show better baseline clinical features, with a lower number of exacerbations (P = 0.04), a better ACT score (P = 0.07), less frequent use of LAMA to control severe asthma symptoms and an overall better lung function, with higher values of FEV_1_ (P < 0.0001), FVC (P < 0.0001) and FEF_25-75_ (P < 0.0001). Interestingly, the improvement of the four elements characterizing CliR is not uniform through follow up timepoints. Indeed, while the reduction in exacerbations, OCS administration and the ACT increase become evident between 3 and 6 months from the start of anti-IL5/IL-5r treatment, the gap in FEV_1_ response to biologic treatment has two spikes, one between 1 and 3 months from baseline and one between 6 and 12 months (**Table 3**). As shown in **Figure 3**, FEV1 improvement is sustained during the follow up only in remittent patients, dropping twice (T1-T3 and T6-T12) before the end of the study in those who did not achieve CliR. These data were also confirmed in our LASSO regression model (**Table 4**), where these changes in main remission items during the follow up resulted consistently associated with CliR development. Previous studies reported that several clinical, functional and biological severe asthma features could be related to biologics clinical response (5, 15, 16). Nevertheless, considering the complexity of severe asthma, we believed that tracing the trajectory of CliR only using baseline features of our cohort would have been less reliable than evaluating the changes of specific clinical and functional items of remission. For this reason, we merged baseline and follow up relevant features in our predictive model, describing how CliR features develop after the start of biologic therapies. The timely improvement in respiratory function and other observed remission features strongly indicate that these therapies have a fast-acting potential in inducing severe asthma CliR. As previously postulated, the path leading to CliR seems to follow a specific timeline since a real difference in main asthma clinical items (exacerbations, OCS, symptoms) between remittent and non-remittent patients becomes clinically visible only after at least 3 months of biologic therapy (17). Moreover, lung function improvement seems to be affected by biologic therapies earlier than other clinical features. As stated by registration trials and real-life evidence on Mepolizumab and Benralizumab (18–23), FEV_1_ improvement is generally reported between 4 and 12 weeks from the first monoclonal antibody administration, which is in line with our findings on the overall population and on patients with CliR. Although we are not aware of the potential mechanism explaining the double gap we found on FEV1 improvement in remittent vs non-remittent patients, it has been reported that blood and sputum eosinophilia could play a role determining lung function trajectory. Patients with a lower sputum eosinophilia, along with a more pronounced neutrophilic airway inflammation, tend to have a worse FEV1 trajectory, while those with a marked blood and sputum eosinophilia have a fast and sustained improvement in lung function (24). Moreover, we cannot also exclude that a certain degree of airway remodeling or the impact of comorbidities in non-remittent patients could have influenced the FEV1 improvement with anti IL5/IL5r therapy.

Among the other predictors included in our Model 1 LASSO regression, CliR is associated with a lower BMI, a greater BEC, a lower number of exacerbations at baseline, the absence of positive skin prick tests, a positive history of CRwNP and with the absence of a previous diagnosis of EGPA, bronchiectasis or osteoporosis. Moreover, patients who did not receive anti-muscarinic therapy but were more frequently treated with their inhaled reliever were also more frequently classified as remittent. Whereas the relationship between CliR, a lower BMI, a lower baseline exacerbation rate, an increased BEC and comorbid CRwNP have been previously identified (5, 8, 10, 15, 25), remains poorly described the impact of other comorbidities on this outcome. In patients suffering from EGPA, the add-on treatment with anti-IL5/IL-5r monoclonal antibodies provides a consistent clinical and functional improvement, allows corticosteroid tapering and favors the achievement of EGPA remission (26–28). Nevertheless, the simultaneous presence of SEA and comorbid EGPA could slow biologic treatment response (29), also influencing the achievement of CliR. Furthermore, complete OCS weaning after biologic administration in patients with EGPA could be challenging, not only due to the disease itself but also from a “cultural” standpoint, since corticosteroid cessation mainly depends on centers experience and confidence with monoclonal antibodies. Similarly to EGPA, biologics targeting IL5/IL5r have shown to improve exacerbation frequency and OCS consumption in patient with SEA and bronchiectasis (30). Nevertheless, the odds for CliR achievement in these patients seems to be lower when compared with those with SEA (31). Possible reasons for this finding could include an increased expression of non-T2 inflammation pathways, severe muco-ciliary disfunction and microbiologic colonization with multi drug resistant microorganism, which could negatively affect the clinical response to biologics (31, 32). As regards osteoporosis, there are few studies investigating its relationship with severe asthma outcomes. Considering the close causal link between osteoporosis and corticosteroid use (33), a worse clinical response to inhaled or systemic steroid-based therapies could induce patients and clinicians to increase the therapeutic corticosteroid dose, increasing the risk for osteoporosis development. This event would give birth to a distorted mechanism, where the lack of response induces the increase of inhaled or systemic steroid dosages, with negative repercussions on bone metabolism and osteopenia.

Another important aspect is the relationship between CliR and the presence of a positive skin prick test. Our results showed a negative impact of the presence of atopy on CliR, as also confirmed by the lower number of patients with an atopic trait in the remittent group (48% vs 60.5%, P = 0.08). As recently reported by Moermans and colleagues, the presence of higher sputum levels of several biomarkers related to eosinophilic inflammation (IL-5, eotaxin-1, eosinophil peroxidase) can predict CliR achievement (16). Moreover, the coexistence of the eosinophilic and the atopic traits in severe asthma patients could lead to the simultaneous activation of several cytokine pathways, whit possible drawbacks on treatment response (34).

As stated before, CliR seems also to be associated with non-biologic treatments such as LAMA and reliever therapy. To date, we are unaware of what possible reason could explain the link between the use of relievers and remission achievement. However, the negative correlation between LAMA use and CliR could be explained by the tendency to prescribe anti-muscarinics in patients with a worse FEV_1_, which are those who will have more difficulty in fulfilling remission criteria. Another important aspect concerns the impact of lung function over severe asthma remission. As also shown in **Table 2**, a better baseline lung function foreshadows CliR achievement, not only in terms of FEV_1_, but also considering FEF_25-75._ The multicentric ATLANTIS study revealed a significant association between small airway disfunction and asthma severity (35). Besides, Chan and colleagues previously highlighted the close relationship between biologic treatment response and small airways functional assessment, addressing FEF_25-75_ and impulse oscillometry (IOS) measures as useful tools for severe asthma management (36, 37). Our data seem to confirm these findings, not only showing a consistent improvement of FEF_25-75_ after 1 year of biologic therapy (see **Supplementary Table 3**), but also certifying its predictive role for CliR achievement (see **Table 4**).

Our study has several limitations, such as its retrospective design, a limited follow up time and the lack of information on other functional (i.e. IOS, bronchial challenge test) and inflammatory biomarkers (i.e. induced sputum), which would have better described what pathophysiological mechanism primary acts in the path toward CliR. Nevertheless, the multicentric nature of the study, a rigorous selection of the enrolled patients and a robust predictive model for CliR allows the generalizability of our results, strengthening the idea of a personalized clinical approach according to patient response to biologic treatments.

In conclusion, our study highlighted a possible identikit for patients achieving CliR after a 1-year course of Mepolizumab or Benralizumab. “Remission potential” seem to be characterized by a specific subset of baseline clinical, functional, and biological features, as well as a timely improvement in all main remission criteria within 24 weeks from biologic treatment start. These results could allow clinicians to better tailor their therapeutic choices in patients with severe asthma, applying the principles of precision medicine to everyday clinical practice.

## Data availability statement

The raw data supporting the conclusions of this article will be made available by the authors, without undue reservation.

## Ethics statement

The studies involving humans were approved by Bari Institutional Ethics Committees. The studies were conducted in accordance with the local legislation and institutional requirements. The participants provided their written informed consent to participate in this study.

## Author contributions

GC: Conceptualization, Project administration, Supervision, Validation, Writing – original draft. AP: Data curation, Formal analysis, Investigation, Methodology, Validation, Visualization, Writing – original draft. SN: Data curation, Methodology, Validation, Writing – original draft. AD: Data curation, Validation, Writing – review & editing. AV: Data curation, Investigation, Project administration, Writing – review & editing. CCa: Data curation, Supervision, Writing – review & editing. CP: Data curation, Supervision, Validation, Writing – review & editing. FM: Data curation, Project administration, Supervision, Visualization, Writing – original draft. GS: Data curation, Validation, Writing – review & editing. GV: Data curation, Validation, Writing – review & editing. MD: Data curation, Supervision, Validation, Writing – review & editing. MC: Data curation, Supervision, Validation, Writing – review & editing. MT: Data curation, Validation, Writing – review & editing. NS: Data curation, Supervision, Validation, Writing – review & editing. CCr: Conceptualization, Data curation, Project administration, Supervision, Validation, Writing – review & editing.
