# Supplementary material for: Features of severe asthma response to anti-IL5/IL5r therapies: identikit of clinical remission
Source: Front Immunol. 2024 Jan 23;15:1343362. doi: 10.3389/fimmu.2024.1343362 (PMC10848329; doi:10.3389/fimmu.2024.1343362)
Supplement: Supplementary file 1 [file DataSheet_1.docx]

Supplementary Material

**Supplemental methods**

Missing data

In our dataset, we found the following missing data:

**Table E1**. Missing data according to follow up time.

| **Variable** | **Missing values (%, n)** | | | | |
| --- | --- | --- | --- | --- | --- |
|  | **T0** | **T1** | **T3** | **T6** | **T12** |
| BMI | 1.9 (5) | *NA* | *NA* | *NA* | *NA* |
| Years from asthma diagnosis | 1.1 (3) | *NA* | *NA* | *NA* | *NA* |
| Age of asthma onset | 1.9 (5) | *NA* | *NA* | *NA* | *NA* |
| OCS mean dose | 3.5 (7) | 5.2 (5) | 4.8 (4) | 4.7 (3) | 13.9 (6) |
| FEV1 (%) | 0 | 18.8 (50) | 18.8 (50) | 13.5 (36) | 0 |
| FEV1 (lt) | 0.4 (1) | 19.2 (51) | 18.8 (50) | 13.5 (36) | 0 |
| FVC (lt) | 3.8 (10) | 19.2 (51) | 19.2 (54) | 17.3 (46) | 3.8 (10) |
| FVC (%) | 3.4 (9) | 19.2 (51) | 19.2 (54) | 17.3 (46) | 3.8 (10) |
| FEV1/FVC | 3.8 (10) | 19.2 (51) | 18.8 (50) | 17.3 (46) | 3.8 (10) |
| FEF 25-75 (%) | 11.3 (30) | 27.4 (73) | 30.1 (80) | 30.5 (81) | 20.3 (54) |
| FeNO | 30.5 (81) | 43.6 (116) | 53 (141) | 45.9 (122) | 44.4 (118) |
| BEC (cell/mcl) | 1.1 (3) | 22.2 (59) | 15.4 (41) | 14.7 (39) | 11.7 (31) |
| Total IgE | 13.9 (37) | *NA* | *NA* | *NA* | *NA* |
| ACT | 0 | 10.5 (28) | 10.9 (29) | 4.9 (13) | 0 |

BMI, Body Mass Index; OCS, Oral Corticosteroids; FEV1, Forced Expiratory Volume; FVC, Forced Vital Capacity; FEF, Forced Expiratory Flow; FeNO, [Fractional Exhaled Nitric Oxide](https://www.bing.com/ck/a?!&&p=f840e81ec40e23ecJmltdHM9MTY5MTc5ODQwMCZpZ3VpZD0wZDZmY2FiYS0yYjdhLTZhYjgtMTJjZi1kYWE5MmExMTZiYWUmaW5zaWQ9NTIyNA&ptn=3&hsh=3&fclid=0d6fcaba-2b7a-6ab8-12cf-daa92a116bae&psq=feno+medicine&u=a1aHR0cHM6Ly93d3cuZW5nbGFuZC5uaHMudWsvYWFjL3doYXQtd2UtZG8vaW5ub3ZhdGlvbi1mb3ItaGVhbHRoY2FyZS1pbmVxdWFsaXRpZXMtcHJvZ3JhbW1lL3JhcGlkLXVwdGFrZS1wcm9kdWN0cy9mcmFjdGlvbmFsLWV4aGFsZWQtbml0cmljLW94aWRlLw&ntb=1); BEC, blood eosinophil count; ACT, Asthma Control Test;

Before starting the imputation process, we performed Little’s test for missing completely at random data.

**Table E2**. Little’s test for missing variables. A p-value > 0.05 was considered as indicative of MCAR data.

| **Little’s MCAR test** | **Follow up** | | | | |
| --- | --- | --- | --- | --- | --- |
|  | **T0** | **T1** | **T3** | **T6** | **T12** |
| Chi^2^ | 63.7 | 66.3 | 48.6 | 49.5 | 16.7 |
| P value | 0.84 | 0.16 | 0.37 | 0.12 | 0.08 |

Then, we used the expectation-maximization imputation method with 25 iterations to replace missing values for every variable. FeNO and FEF_25-75_ had a higher number of missing data, so we did not feel confident that an imputation analysis would be the most suitable approach for these two variables. For this reason, we decided to impute only FEF_25-75_ T0 and T12 values for the analysis, which had a lower number of missing data (10.8% and 19.4%, respectively).

Regression analysis

To explore which factors could be predictive of the achievement of CliR, we run a multivariate logistic least absolute shrinkage and selection operator (LASSO) regression, selecting variables significantly associated with the outcome. LASSO regression, differently from other regression analyses, allows variables selection using a penalty (λ), which reduces variance and shrinks towards zero non-relevant covariates (Zou, 2012). Accordingly, LASSO regression avoids overfitting and multicollinearity issues, providing a consistent panel of independent variables associated with the desired outcome. Considering the high number of FeNO missing values, we decided to perform 2 separate regression analysis. The first model (Model 1) accounts most important clinical, functional and biological recorded variables (see Table 4) without considering FeNO. The second model (Model 2) also includes FeNO, excluding missing values from the analysis. Main results from LASSO analysis were described considering Model 1, which encompasses the whole enrolled population, while Model 2 was used to rule out/in FeNO as a pivotal covariate influencing remission achievement. For the variable “reliever use” (see Table 4), we considered only the as-need self-administration of SABA or ICS-Formoterol. Patients were asked “how many times a day you used your reliever therapy in the last month?”, reporting the answer as a continuous variable for our analysis. Patients who underwent a previous treatment with monoclonal antibodies were excluded from the analysis. Before running the analysis, non-binary data were scaled using the function “rescale” from R software (version 4.0.2, R Foundation). Then, we performed LASSO regression analysis using “glmnet” package from R software (version 4.0.2, R Foundation). Finally, we tested the robustness of our models using receiver operating characteristics (ROC) curves with their areas under the curves (AUC). The first ROC curve with AUC was calculated using the complete dataset for both Model 1 and Model 2. Then, considering the risk of an unrepresentative data sample due to the small size of the test dataset, we further added to our ROC curves the AUC considering not only the overall model but also the training dataset. A P value < 0.05 was considered as statistically significant.

**e-Figure legend**

**Figure E1**. Changes of exacerbations frequency, access to emergency departments, OCS use and good control of asthma symptoms (ACT ≥ 20) after 1 year of biologic treatment according to follow up timepoints. * P<0.05; **P<0.01; *** P<0.001; **** P<0.0001

**Figure E2**. Functional differences at A) baseline and B) T12 according to clinical remission achievement. * P<0.05; **P<0.01; *** P<0.001; **** P<0.0001

**References**

Zou, H. (2012). The Adaptive Lasso and Its Oracle Properties. *Https://Doi.Org/10.1198/016214506000000735*, *101*(476), 1418–1429. https://doi.org/10.1198/016214506000000735
