# Supplementary material for: Features of severe asthma response to anti-IL5/IL5r therapies: identikit of clinical remission
Source: Front Immunol. 2024 Jan 23;15:1343362. doi: 10.3389/fimmu.2024.1343362 (PMC10848329; doi:10.3389/fimmu.2024.1343362)
Supplement: Supplementary file 2 [file Table_3.docx]

**Table E3.** Characteristics of severe asthma patients treated with anti-IL5/IL5r according to follow-up time

|  | **T0** | **T1** | **T3** | **T6** | **T12** | **P Value** |
| --- | --- | --- | --- | --- | --- | --- |
| Exacerbations (%, n)  Exacerbations at 1^st^ visit (Median, IQR)  Access to ED (%, n) | 96.2 (256)  4 [3˗6]  31.6 (84) | 13.2 (35)  0 [0-0]  0.4 (1) | 13.2 (35)  0 [0-0]  1.9 (5) | 21.1 (56)  0 [0-0]  1.1 (3) | 20.7 (55)  0 [0-0]  1.9 (5) | **<0.0001**  **<0.0001**  **<0.0001** |
| Asthma treatment   - LAMA (%, n) - Reliever (%, n) - Reliever use (Median, IQR#) - LTRA (%, n) - OCS (%, n) - OCS dose at baseline (Median, IQR) | 69.9 (186)  59 (157)  1 [0-3]  44 (118)  75.6 (201)  12,5 [5-25] | 69.5 (185)  18.4 (49)  0 [0-0]  42.5 (113)  36.1 (96)  5.5 [5-15] | 69.9 (186)  14.3 (38)  0 [0-0]  39.5 (105)  31.6 (84)  5 [3.2-12.5] | 68.4 (174)  8.3 (22)  0 [0-0]  39.5 (105)  24.1 (64)  5 [3-12.5] | 65.4 (174)  10.5 (28)  0 [0-0]  39.1 (104)  16.2 (43)  5.6 [4-12.5] | **<0.0001**  **<0.0001**  0.07  **<0.0001**  **<0.0001** |
| - ACT baseline (Mean, SD) | 13.4 ± 4 | 18.7 ± 3.6 | 20.7 ± 3.2 | 20.9 ± 3.4 | 21.3 ± 3.3 | **<0.0001** |
| Lung function   - FEV1 (%, Mean, SD) - FEV1 (lt, Mean, SD) - FVC (%, Mean, SD) - FVC (lt, Mean, SD) - FEV1/FVC (Mean, SD) - FEF_25-75_ (Median, IQR) | 69.8 ± 22.1  1.9 ± 0.8  84.5 ± 20.5  2.8 ± 1  67 ± 12.3  39 [25-56.2] | 76.3 ± 19.9  2.1 ± 0.7  88 ± 18  3 ± 0.9  68.2 ± 13.7  45 [31.5-61] | 78.1 ± 19.8  2.1 ± 0.8  90.3 ± 16.1  3 ± 0.9  71.5 ± 13.5  50 [35-68.] | 80.7 ± 19.6  2.2 ± 0.8  92.1 ± 16.8  3 ±0.9  71.7 ± 14.1  53 [32-70] | 82.3 ± 21.4  2.2 ±0.8  93.4 ± 18.8  3 ± 1.1  72.8 ± 15.8  57.9 [41.7-71] | **<0.0001**  **<0.0001**  **<0.0001**  **0.0004**  **<0.0001**  **<0.0001** |
| FeNO (ppb, Median, IQR)  BEC (cells/mcl, Median, IQR) | 36 [19.4-59.5]  645 [420-920] | 28 [18.6-52]  82.5 [0-119] | 23 [12-43]  40 [0-80] | 25 [16.5-40]  40 [0-76] | 23 [16-40]  40 [0-80] | **0.0007**  **<0.0001** |

IQR, InterQuartile Range; ED, Emergency Department; LAMA, Long-Acting Muscarinic Antagonists; LTRA, Leukotriene receptor antagonist therapy; OCS, Oral Corticosteroids; ACT, Asthma Control Test; SD, Standard Deviation; BD, Bronchodilator; FEV1, Forced Expiratory Volume; FVC, Forced Vital Capacity; FEF, Forced Expiratory Flow; FeNO, [Fractional Exhaled Nitric Oxide](https://www.bing.com/ck/a?!&&p=f840e81ec40e23ecJmltdHM9MTY5MTc5ODQwMCZpZ3VpZD0wZDZmY2FiYS0yYjdhLTZhYjgtMTJjZi1kYWE5MmExMTZiYWUmaW5zaWQ9NTIyNA&ptn=3&hsh=3&fclid=0d6fcaba-2b7a-6ab8-12cf-daa92a116bae&psq=feno+medicine&u=a1aHR0cHM6Ly93d3cuZW5nbGFuZC5uaHMudWsvYWFjL3doYXQtd2UtZG8vaW5ub3ZhdGlvbi1mb3ItaGVhbHRoY2FyZS1pbmVxdWFsaXRpZXMtcHJvZ3JhbW1lL3JhcGlkLXVwdGFrZS1wcm9kdWN0cy9mcmFjdGlvbmFsLWV4aGFsZWQtbml0cmljLW94aWRlLw&ntb=1); BEC, blood eosinophil count;
