# Supplementary material for: Features of severe asthma response to anti-IL5/IL5r therapies: identikit of clinical remission
Source: Front Immunol. 2024 Jan 23;15:1343362. doi: 10.3389/fimmu.2024.1343362 (PMC10848329; doi:10.3389/fimmu.2024.1343362)
Supplement: Supplementary file 3 [file Table_4.docx]

**Table E4.** Distribution of remission items in the overall population and in patients not achieving clinical remission (CliR),

|  | **Enrolled population** | | **Non-CliR** |
| --- | --- | --- | --- |
| Patients (n) | 266 | 185 | |
| No OCS | 83.8 (223) | 76.8 (142) | |
| No exacerbations | 57.9 (154) | 39.5 (73) | |
| ACT ≥ 20 | 78.9 (210) | 69.7 (129) | |
| FEV1 ≥ 80% | 53.4 (142) | 33 (61) | |
| ≥ 1 item (%, n) | 94.7 (252) | 92.4 (171) | |
| ≥ 2 items (%, n) | 84.2 (224) | 77.3 (143) | |
| ≥ 3 items (%, n) | 64.7 (172) | 49.2 (91) | |
| 4 items (%, n) | 30.4 (81) | 0 | |

CliR, Clinical Remission; OCS, Oral Corticosteroids; FEV1, Forced Expiratory Volume; ACT, Asthma Control Test
