# Supplementary material for: Features of severe asthma response to anti-IL5/IL5r therapies: identikit of clinical remission
Source: Front Immunol. 2024 Jan 23;15:1343362. doi: 10.3389/fimmu.2024.1343362 (PMC10848329; doi:10.3389/fimmu.2024.1343362)
Supplement: Supplementary file 4 [file Table_5.docx]

**Table E5.** Baseline features in patients achieving remission vs non-remittent patients according to the administered biologic therapy.


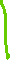


|  | **CliR** | | **P-Value** | **Non-CliR** | | **P-Value** |
| --- | --- | --- | --- | --- | --- | --- |
| Biologic therapy | Mepolizumab | Benralizumab |  | Mepolizumab | Benralizumab |  |
| Patients (%, n) | 55.6 (45) | 44.4 (36) | 0.59 | 59.4 (110) | 40.6 (75) | 0.59 |
| Age (Years, Median, IQR) | 58 [51-64] | 53.5 [45-62.5] | 0.4 | 59 [49.7-65.2] | 57 [46-65] | 0.24 |
| Gender (Male/Female, %) | 37.8/62.2 | 38.9/61.1 | 0.99 | 31.8/68.2 | 36/64 | 0.63 |
| BMI (Days, Mean, IQR) | 25.6 [23.4-27.6] | 25.1 [22.5-27.9] | 0.48 | 27.7 [23.8-30.8] | 26 [23-28.1] | **0.03** |
| Smoke habits (%, n)   - Current smoker - Former smoker - No smoker | 0  37.8 (17)  62.2 (28) | 2.8 (1) 19.4 (7)  77.8 (28) | 0.11 | 7.3 (8)  28.2 (31)  64.5 (71) | 8 (6)  25.3 (19)  66.7 (50) | 0.89 |
| Age of asthma onset (Years, Median, IQR)  Time from asthma diagnosis (Years, Median, IQR)  Positive skin prick test (%, n) | 35 [25-45.5]  17 [6.5-26.5]  53.3 (24) | 37.5 [24.5-40.7]  20 [11.2-28.7]  41.7 (15) | 0.77  0.49  0.37 | 38.5 [24.2-49.2]  20 [10-30]  66.4 (73) | 36 [23-46]  18 [13-30]  52 (39) | 0.45  0.69  0.07 |
| Comorbidities (%, n)   - EGPA - CRwNP - Bronchiectasis - GERD - OSAS - Depression - Urticaria - Atopic dermatitis - Osteoporosis | 11.1 (5)  51.1 (23)  8.9 (4)  28.9 (13)  6.7 (3)  13.3 (6)  0  2.2 (1)  2.2 (1) | 0  66.7 (24)  19.4 (7) 44.4 (16)  0  11.1 (4)  5.6 (2)  8.3 (3)  11.1 (4) | 0.06  0.18  0.2  0.17  0.25  0.99  0.19  0.32  0.17 | 4.5 (5)  45.4 (50)  23.6 (26)  33.6 (37)  8.2 (9)  23.6 (26)  4.5 (5)  3.6 (4)  12.7 (14) | 6.7 (5)  48 (36)  17.3 (13)  37.3 (28)  8 (6)  14.7 (11)  6.7 (5)  6.7 (5)  13.3 (10) | 0.53  0.88  0.36  0.64  0.99  0.19  0.53  0.49  0.99 |
| Exacerbations (%, n)  Exacerbations at 1^st^ visit (Median, IQR)  Access to ED (%, n)  ACT baseline (Mean, SD) | 93.3 (42)  4 [3-6]  31.1 (14)  14.1 ± 4.2 | 97.2 (35)  4 [3-6]  19.4 (7)  14.2 ± 4.5 | 0.62  0.81  0.31  0.87 | 97.3 (78)  5 [3-8]  29.1 (32)  12.7 ± 3.9 | 96 (72)  4 [3-6]  41.3 (31)  13.8 ± 3.8 | 0.69  **0.03**  0.11  0.07 |
| Asthma treatment   - LAMA (%, n) - Reliever (%, n) - Reliever use (Median, IQR#) - LTRA (%, n) - OCS (%, n) - OCS dose at baseline (Median, IQR) | 53.3 (24)  55.6 (25)  1 [0-4]  44.6 (25)  64.4 (29)  13.9 [6.4-25] | 66.7 (24)  61.1 (22)  1.5 [0-2]  27.8 (10)  75 (10)  12.5 [5-25] | 0.26  0.66  0.43  **0.01**  0.34  0.2 | 70 (77)  60 (66)  1 [0-3]  45.5 (50)  78.2 (86)  12.5 [6.2-25] | 81.3 (61)  58.7 (44)  2 [0-3]  44 (33)  78.7 (59)  10 [5-25] | 0.09  0.88  0.88  0.88  0.99  **0.03** |
| Lung function   - FEV_1_ (%, Mean, SD) - FEV_1_ (lt, Mean, SD) - FVC (%, Mean, SD) - FVC (lt, Mean, SD) - FEV1/FVC (Mean, SD) - FEF_25-75_ (Median, IQR) | 79.4 ± 17  2.2 ±0.8  90.6 ± 15.1  3.1 ± 1  71.3 ±12.6  54.6 [41-62.5] | 78.9 ± 24.6  2.2 ± 0.9  93.1 ± 21.9  3.2 ± 1.2  68.5 ± 12.2  48 [31.2-66.5] | 0.91  0.89  0.54  0.82  0.31  0.15 | 67.5 ± 23.5  1.7 ± 0.8  83.6 ± 22.2  2.6 ± 1  65.4 ± 12  32 [19-50.1] | 63.2 ± 18.2  1.8 ± 0.6  78.2 ± 17.8  2.7 ± 0.8  66 ± 12.1  34 [24-49] | 0.18  0.78  0.08  0.71  0.74  0.57 |
| FeNO (ppb, Median, IQR)  BEC (cells/mcl, Median, IQR)  Total IgE ( IU/mL , Median, IQR) | 41 [22.2-66.2]  560 [410-1080]  181 [38-344.1] | 32 [24-57]  665 [512.5-890]  178.5 [76-335.4] | 0.58  0.4  0.61 | 32 [11.4-49.2]  623.5 [400-900]  173 [66.1-352] | 41.5 [24.5-61.2]  680 [415-910]  112 [54-295] | **0.02**  0.68  0.34 |

IQR, Interquartile Range; EGPA, Eosinophilic Granulomatosis with Polyangiitis; CRwNP, Chronic Rhinosinusitis with Nasal Polyps; BMI, Body Mass Index; ED, Emergency Department; OCS, Oral Corticosteroids; GERD, [Gastroesophageal reflux disease](https://www.bing.com/ck/a?!&&p=0ca6fd4dfa61bd3aJmltdHM9MTY5MTc5ODQwMCZpZ3VpZD0wZDZmY2FiYS0yYjdhLTZhYjgtMTJjZi1kYWE5MmExMTZiYWUmaW5zaWQ9NTIzMQ&ptn=3&hsh=3&fclid=0d6fcaba-2b7a-6ab8-12cf-daa92a116bae&psq=GERD+MEDICINE&u=a1aHR0cHM6Ly93d3cubWF5b2NsaW5pYy5vcmcvZGlzZWFzZXMtY29uZGl0aW9ucy9nZXJkL2RpYWdub3Npcy10cmVhdG1lbnQvZHJjLTIwMzYxOTU5&ntb=1); OSAS, Obstructive Sleep Apnea Syndrome; ED, Emergency Department; ACT, Asthma Control Test; LAMA, Long-Acting Muscarinic Antagonists; LTRA, Leukotriene receptor antagonist therapy; SD, Standard Deviation; FEV_1_, Forced Expiratory Volume; FVC, Forced Vital Capacity; FEF, Forced Expiratory Flow; FeNO, [Fractional Exhaled Nitric Oxide](https://www.bing.com/ck/a?!&&p=f840e81ec40e23ecJmltdHM9MTY5MTc5ODQwMCZpZ3VpZD0wZDZmY2FiYS0yYjdhLTZhYjgtMTJjZi1kYWE5MmExMTZiYWUmaW5zaWQ9NTIyNA&ptn=3&hsh=3&fclid=0d6fcaba-2b7a-6ab8-12cf-daa92a116bae&psq=feno+medicine&u=a1aHR0cHM6Ly93d3cuZW5nbGFuZC5uaHMudWsvYWFjL3doYXQtd2UtZG8vaW5ub3ZhdGlvbi1mb3ItaGVhbHRoY2FyZS1pbmVxdWFsaXRpZXMtcHJvZ3JhbW1lL3JhcGlkLXVwdGFrZS1wcm9kdWN0cy9mcmFjdGlvbmFsLWV4aGFsZWQtbml0cmljLW94aWRlLw&ntb=1); BEC, blood eosinophil count
