# Supplementary material for: Features of severe asthma response to anti-IL5/IL5r therapies: identikit of clinical remission
Source: Front Immunol. 2024 Jan 23;15:1343362. doi: 10.3389/fimmu.2024.1343362 (PMC10848329; doi:10.3389/fimmu.2024.1343362)
Supplement: Supplementary file 5 [file Table_6.docx]

**Table E6**. Features of patients with severe asthma after 12 months of anti-IL5/IL5r therapy according to clinical remission (CliR) achievement.

|  | **CliR** | **Non-CliR** | **P value** |
| --- | --- | --- | --- |
| Patients (%, n) | 30.5 (81) | 69.5 (185) |  |
| Exacerbations/year (%, n)  Exacerbations (Median, IQR)  Access to ED (%, n)  ACT (Mean, SD) | 0  0  0  23.2 ± 1.8 | 29.7 (55)  0 [0-1]  2.7 (5)  20.5 ± 3.5 | **<0.0001**  **<0.0001**  0.33  **<0.0001** |
| Asthma treatment   - LAMA (%, n) - Reliever use (%, n) - LTRA (%, n) - OCS (%, n) - OCS dose at baseline (Median, IQR) | 55.6 (45)  3.7 (3)  31 (38.3)  0  0 | 69.7 (129)  13.5 (25)  39.5 (73)  23.2 (43)  5.6 [4-12.5] | **0.03**  **0.02**  0.89  **<0.0001**  **<0.0001** |
| Lung function   - FEV1 (%, Mean, SD) - FEV1 (lt, Mean, SD) - FVC (%, Mean, SD) - FVC (lt, Mean, SD) - FEV1/FVC (Mean, SD) - FEF_25-75_ (Median, IQR) | 99.1 ± 13.3  2.6 ± 0.8  106.1 ± 14.1  3.4 ± 1.1  77.4 ± 14.9  67 [56.3-79] | 74.9 ± 20.1  2 ± 0.78  87.9 ± 17.9  2.8 ± 1  70.9 ± 15.8  52 [33.5-67.4] | **<0.0001**  **<0.0001**  **<0.0001**  **<0.0001**  **0.002**  **<0.0001** |
| FeNO (ppb, Median, IQR)  BEC (cells/mcl, Median, IQR) | 21 [18-44]  30 [0-85] | 24 [15-35]  40 [0-80] | 0.95  0.73 |

CliR, Clinical Remission; IQR, Interquartile Range; ED, Emergency Department; ACT, Asthma Control Test; LAMA, Long-Acting Muscarinic Antagonists; LTRA, Leukotriene receptor antagonist therapy; OCS, Oral Corticosteroids; BD, Bronchodilator; SD, Standard Deviation; FEV1, Forced Expiratory Volume; FVC, Forced Vital Capacity; FEF, Forced Expiratory Flow; FeNO, [Fractional Exhaled Nitric Oxide](https://www.bing.com/ck/a?!&&p=f840e81ec40e23ecJmltdHM9MTY5MTc5ODQwMCZpZ3VpZD0wZDZmY2FiYS0yYjdhLTZhYjgtMTJjZi1kYWE5MmExMTZiYWUmaW5zaWQ9NTIyNA&ptn=3&hsh=3&fclid=0d6fcaba-2b7a-6ab8-12cf-daa92a116bae&psq=feno+medicine&u=a1aHR0cHM6Ly93d3cuZW5nbGFuZC5uaHMudWsvYWFjL3doYXQtd2UtZG8vaW5ub3ZhdGlvbi1mb3ItaGVhbHRoY2FyZS1pbmVxdWFsaXRpZXMtcHJvZ3JhbW1lL3JhcGlkLXVwdGFrZS1wcm9kdWN0cy9mcmFjdGlvbmFsLWV4aGFsZWQtbml0cmljLW94aWRlLw&ntb=1); BEC, blood eosinophil count
