# Supplementary material for: Features of severe asthma response to anti-IL5/IL5r therapies: identikit of clinical remission
Source: Front Immunol. 2024 Jan 23;15:1343362. doi: 10.3389/fimmu.2024.1343362 (PMC10848329; doi:10.3389/fimmu.2024.1343362)
Supplement: Supplementary file 6 [file Table_7.docx]

**Table E7.** Receiver Operating Characteristic (ROC) curves of multivariate LASSO regression models

|  | **N. of values** | **AUC** | **95% CI** | **P-Value** |
| --- | --- | --- | --- | --- |
| ***LASSO logistic regression model*** |  |  |  |  |
| Model 1 | 266 | 0.87 | [0.83-0.91] | **<0.0001** |
| Model 2 | 185 | 0.88 | [0.83-0.93] | **<0.0001** |

LASSO, Least Absolute Shrinkage and Selection Operator; AUC, Area Under the Curve; CI, Confidence Interval
