# Supplementary figures and images for: Features of severe asthma response to anti-IL5/IL5r therapies: identikit of clinical remission
Source: Front Immunol. 2024 Jan 23;15:1343362. doi: 10.3389/fimmu.2024.1343362 (PMC10848329; doi:10.3389/fimmu.2024.1343362)

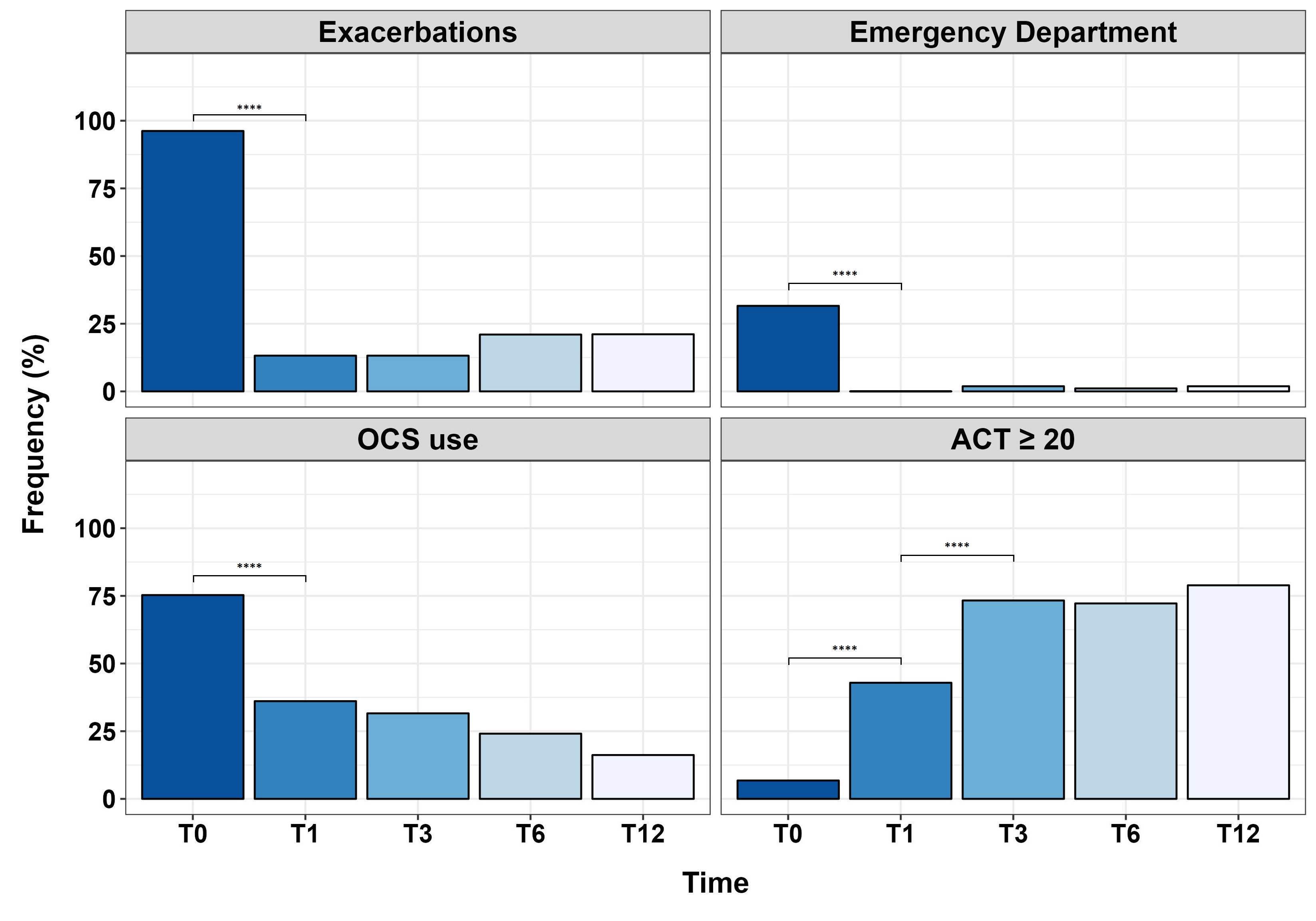

Supplement: Supplementary file 7 [file Image_1.jpeg]

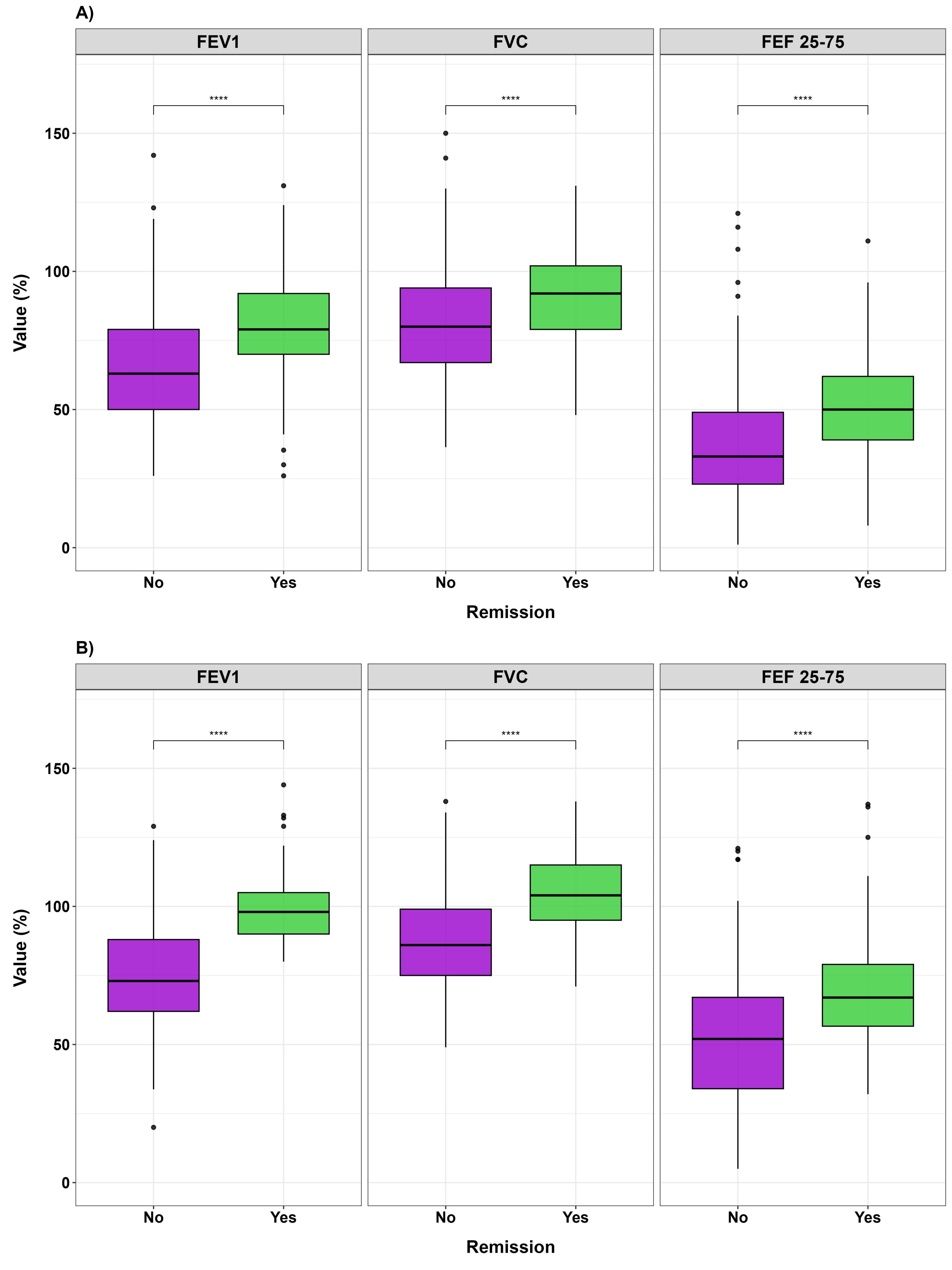

Supplement: Supplementary file 8 [file Image_2.jpeg]
